# Efficacy of the hemostatic device VasoSTAT and the study of hemostatic factor

**DOI:** 10.1038/s41598-021-00892-5

**Published:** 2021-11-01

**Authors:** Hirokazu Naganawa, Akira Ito, Shinrou Saiki, Daisuke Nishi, Shinichi Takamatsu, Yoshihisa Ito, Takeshi Suzuki

**Affiliations:** Department of Cardiology, Toyokawa City Hospital, 23 Nozi Yawata-cho, Toyokawa City, Aichi, 442-8561 Japan

**Keywords:** Cardiology, Medical research

## Abstract

Recently, trans-radial intervention has gained popularity as a common procedure to reduce hemorrhagic complications. However, the cuff-type hemostatic device (TR Band) previously used at our institution required 6 h to achieve hemostasis. Since July 2016, we have been using the VasoSTAT, a new hemostatic device that could achieve hemostasis in 4 h. In a verification study, we found that prolonged activated clotting time (ACT) was related to transient hemorrhage occurrence after the hemostatic procedure. Therefore, we designed a hemostatic protocol based on ACT and evaluated its efficacy. In this retrospective and observational study, 78 and 111 patients used the VasoSTAT and TR Band, respectively, from July 2015 to May 2017. In the VasoSTAT group, the ACTs were significantly lower in the hemostasis success (246 ± 46 s) than in the failure group patients (327 ± 59 s) (*P* < 0.01). Therefore, we applied the hemostatic protocol to 271 patients from May 2017 to March 2020. The hemostasis success rate was 96% in the post-protocol applied group patients, which was significantly higher than the 82% success rate in the pre-protocol applied group patients (*P* < 0.01). VasoSTAT resulted in adequate hemostasis in 4 h. Further, ACT was predictive of adequate hemostasis.

## Introduction

In recent years, trans-radial intervention (TRI) has become a common procedure due to reduced access site bleeding and hemorrhagic complications, decreased time to ambulation, improved overall survival in patients with ST-segment elevation myocardial infarction, and increased patient comfort, compared with trans-femoral intervention^1–7^. Our institution previously used the cuff-type hemostatic device, TR Band (Terumo Medical, Tokyo, Japan); however, it required 6 h to achieve hemostasis. The VasoSTAT (Forge Medical, Bethlehem, Pennsylvania, USA), which is another hemostatic device, can shorten hemostasis time to 4 h. Although the TR Band presses a wide area of the puncture site, including the surrounding vessels and nerves with its bladder, the VasoSTAT restrictively presses the puncture site by its convex and oval surfaces, with minimal pressure. Owing to these mechanisms and features, the VasoSTAT can reduce pain and numbness and shorten hemostasis time by maintaining the puncture site circulation compared with the TR Band. Hence, the primary purpose of this study was to compare the efficacy of the two aforementioned hemostasis devices. As there is a paucity of current literature discussing factors affecting hemostasis success rates, this study also aims to investigate these factors and the efficacy of the hemostatic protocol we designed based on them.

## Methods

We studied 460 patients who underwent percutaneous coronary intervention (PCI) by TRI at our hospital, and used either the VasoSTAT or TR Band as a hemostasis device from June 2015 to March 2020. The patients with an intra-aortic balloon pumping and/or a percutaneous cardiopulmonary support were excluded. We began using the VasoSTAT in July 2016. The hemostasis time was 6 h using the TR Band, and 4 h using the VasoSTAT. All patients were initially administered 8000 units of heparin, which was appropriately titrated during the PCI procedure. In the TR Band group patients, the band was applied at the arterial puncture site, the bladder was inflated with 10 mL of air, and the sheath was pulled under the band. In the VasoSTAT group patients, the wing and pad were applied at the arterial puncture site, the plunger was compressed, and the sheath was pulled under the plunger. When we pulled the sheath, we measured the activated clotting time (ACT) by the test tube method, using the Hemochron Response device (Accriva Diagnostics, San Diego, California, USA). We observed the puncture site after 15 min, 30 min, 1 h, 2 h, and 4 h in the VasoSTAT group and 15 min, 30 min, 1 h, 2 h, 4 h, and 6 h in the TR Band group. We defined hemostatic failure as a requirement of an additional hemostasis time of more than 5 min and/or a necessity for different hemostatic devices.

All statistical analyses were performed using EZR (Saitama Medical Center, Jichi Medical University, Saitama, Japan), which is a graphical user interface for R (The R Foundation for Statistical Computing, Vienna, Austria). Continuous variables are expressed as mean and standard deviation. For continuous data, groups of normally distributed and homoscedastic variables were compared using the Student’s t test, normally distributed and heteroscedastic variables were compared using the Welch test, and non-normally distributed variables were compared using the Mann–Whitney U test. Categorical variables are presented as numbers and percentages. Among categorical data, groups were compared using the Chi-squared test. The optimal cutoff for ACT was calculated using the receiver operating characteristic curve (ROC) and determined using the Youden index. A two-tailed P value < 0.05 was considered statistically significant.

All procedures were in accordance with the ethical standards of the institutional and/or national research committee and with the 1964 Helsinki declaration and its later amendments or comparable ethical standards. This study was approved by the institutional review boards (IRB) of Toyokawa City Hospital. Informed consent was obtained from all individual participants included in the study.

## Results

From July 2015 to May 2017, 78 and 111 patients were treated with the VasoSTAT and TR Band, respectively. There were no significant differences in the patient backgrounds between the two groups. The ACTs were not significantly different between the VasoSTAT (261 ± 58 s) and TR Band group patients (269 ± 56 s) (*P* = 0.34). Similarly, the hemostasis success rate was not significantly different between the VasoSTAT (82%) and TR Band group patients (85%) (*P* = 0.31). The rate of 30-day radial artery occlusion (RAO) was lower in the VasoSTAT (0.6%) than in the TR Band group (4.5%) (*P* < 0.01).

The 78 patients that used the VasoSTAT were divided into the hemostasis success and failure groups. Patient characteristics are shown in Table 1. Baseline characteristics, procedural characteristics, antithrombotic therapy, and sheath size were similar in both groups. ACTs were significantly lower in the hemostasis success (246 ± 46 s) than in the failure group patients (327 ± 59 s) (*P* < 0.01). Therefore, based on this result, we designed a hemostasis protocol, based on ACTs.

**Table 1:** Patient characteristics.

Characteristics	Success (n = 64)	Failure (n = 14)	*P* value
Age-years	67.2 (± 11.0)	73.7 (± 12.8)	0.06
Male sex-no. (%)	55 (85.9%)	9 (64.3%)	0.06
Weight-kg	65.3 (± 11.4)	60.1 (± 16.9)	0.31
Body surface area-m^2^	1.70 (± 0.17)	1.60 (± 0.28)	0.22
Body mass index	24.6 (± 3.8)	23.9 (± 3.7)	0.50
Diabetes mellitus-no. (%)	25 (39.1%)	3 (21.4%)	0.21
Hypertension-no. (%)	47 (73.4%)	7 (50.0%)	0.09
Dyslipidemia-no. (%)	42 (65.6%)	9 (64.3%)	0.92
Current smoking-no. (%)	14 (21.9%)	0 (0%)	0.06
Activated Clotting Time-sec	246.3 (± 46,2)	327.4 (± 58.5)	< 0.01*
Acute coronary syndrome-no. (%)	29 (45.3%)	5 (35.7%)	0.51
Emergency-no(%)	25 (39.1%)	4 (28.6%)	0.46
Antithrombotic therapy			
None-no. (%)	25 (39.1%)	4 (28.6%)	0.40
Single-no. (%)	6 (9.4%)	1 (7.1%)	
Aspirin-no. (%)	5 (7.8%)	1 (7.1%)	
Clopidogrel-no. (%)	1 (1.6%)	0 (0.0%)	
Dual-no. (%)	33 (51.5%)	9 (64.3%)	
Aspirin + prasugrel-no. (%)	32 (50.0%)	9 (64.3%)	
Aspirin + clopidogrel-no. (%)	1 (1.6%)	0 (0.0%)	
Sheath size-french			
4-no. (%)	9 (14.1%)	1 (7.1%)	0.83
4.5-no. (%)	2 (3.1%)	1 (7.1%)	
5-no. (%)	48 (75.0%)	11 (78.7%)	
6-no. (%)	5 (7.8%)	1 (7.1%)	

Continuous variables are expressed as mean and standard deviation. Categorical variables are presented as numbers and percentages.

*Significant.

**Figure 1:**
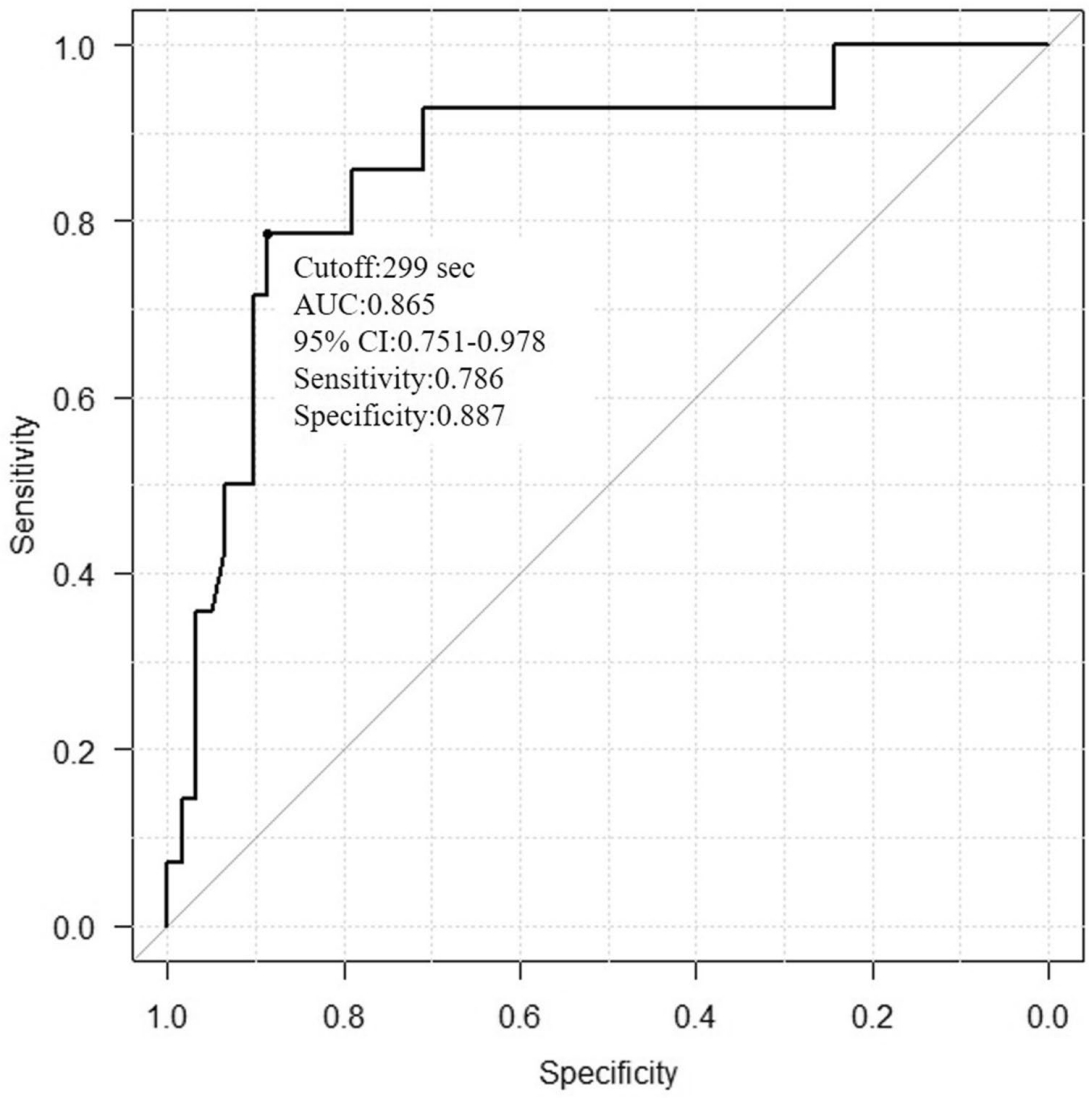
The patients who used the VasoSTAT device were divided into the hemostasis success and failure groups, and the receiver operating characteristic curve was obtained for the activated clotting time (ACT). The cutoff point for ACT was 299 s determined by the Youden index. *AUC* area under the curve, *CI* confidence interval.

The ROC curve is shown in Fig. 1. The cutoff for ACT, sensitivity, and specificity, were 299 s, 0.786, and 0.887, respectively. Considering this result, the cutoff value for ACT was set to 300 s for simplicity. When the ACT was more than 300 s, we neutralized heparin using protamine, as per the protocol (shown in Table 2).

**Table 2:** Protocol.

Activated Clotting Time-sec	Protamine-ml
~ 299	0
300 ~ 349	3
350 ~ 399	5
400 ~	8

**Figure 2:**
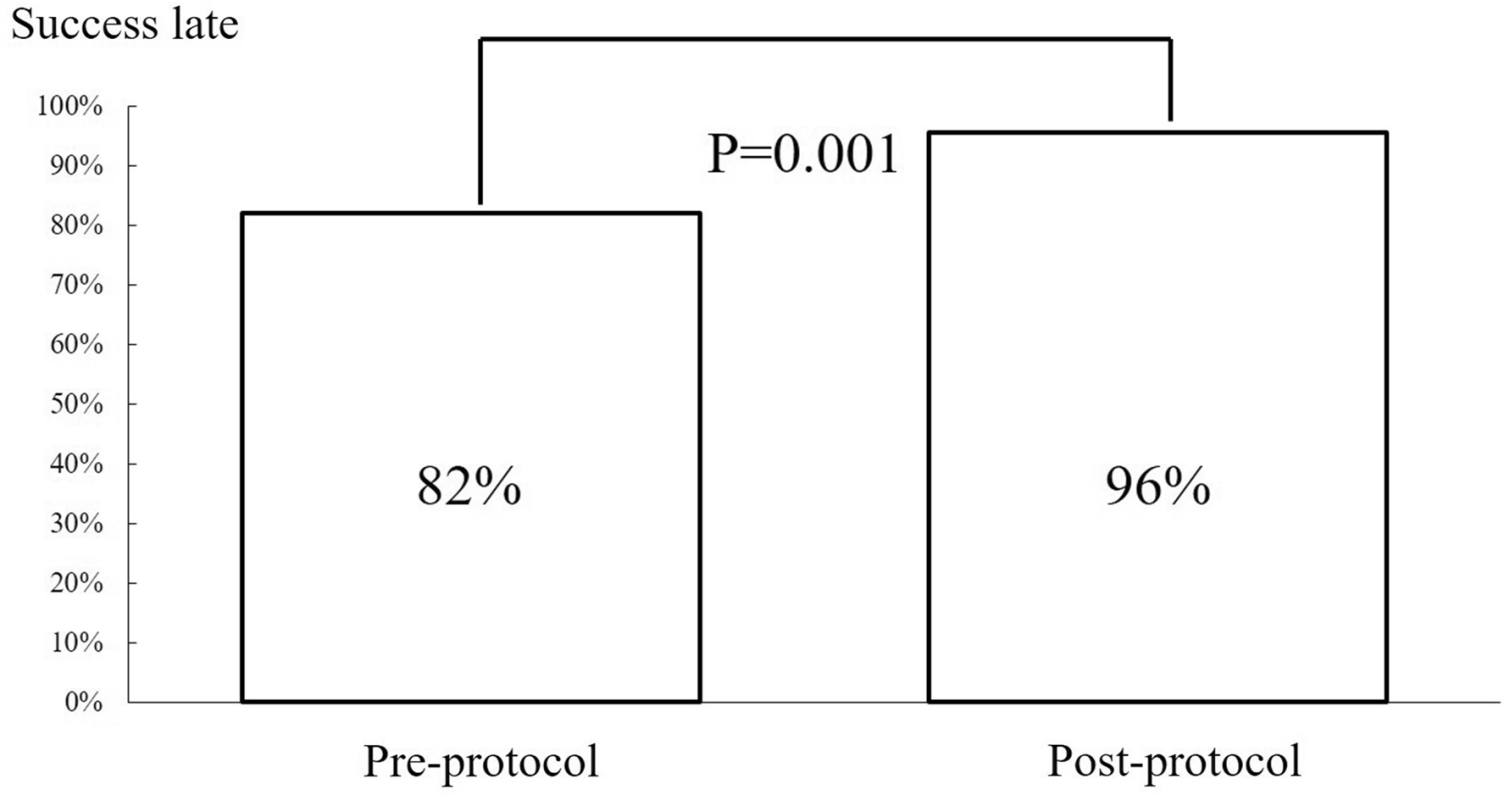
Success rates of pre-protocol and post-protocol groups.

From May 2017 to March 2020, 271 patients were treated with the protocol. Patient characteristics are shown in Table 3. Baseline characteristics, procedural characteristics, antithrombotic therapy, sheath size, and ACT were similar in both groups. The hemostasis success rate was significantly higher in the post-protocol applied group (96%) than in the pre-protocol applied group (82%) (*P* < 0.01). The results are shown in Fig. 2.

**Table 3:** Patient characteristics.

Characteristics	Pre-protocol (n = 78)	Post-protocol (n = 271)	*P* value
Age-years	68.4 (± 11.6)	67.1 (± 11.8)	0.42
Male sex-no. (%)	64 (82.1%)	221 (81.5%)	0.92
Weight-kg	64.7 (± 13.5)	65.2 (± 13.0)	0.74
Body surface area-m^2^	1.68 (± 0.20)	1.70 (± 0.19)	0.43
Body mass index	24.5 (± 4.02)	24.3 (± 3.76)	0.54
Diabetes mellitus-no. (%)	28 (35.9%)	104 (38.4%)	0.69
Hypertension-no. (%)	54 (69.2%)	186 (68.6%)	0.92
Dyslipidemia-no. (%)	51 (65.4%)	179 (66.1%)	0.91
Current smoking-no. (%)	14 (18.0%)	69 (25.5%)	0.17
Activated Clotting Time-sec	261.3 (± 57.7)	255.8 (± 54.9)	0.45
Acute coronary syndrome-no. (%)	34 (43.6%)	132 (48.7%)	0.43
Emergency-no. (%)	29 (37.2%)	116 (42.8%)	0.37
Antithrombotic therapy			
None-no. (%)	29 (37.2%)	79 (29.2%)	0.15
Single-no. (%)	7 (9.0%)	21 (7.7%)	
Aspirin-no. (%)	6 (7.7%)	12 (4.4%)	
Clopidogrel-no. (%)	1 (1.3%)	7 (2.6%)	
DOAC-no. (%)	0 (0.0%)	2 (0.7%)	
Dual-no. (%)	42 (53.8%)	171 (63.1%)	
Aspirin + prasugrel	39 (50.0%)	161 (59.4%)	
Aspirin + cropidogrel	3 (3.8%)	8 (3.0%)	
Aspirin + DOAC	0 (0.0%)	2 (0.7%)	
Sheath size-french			
4-no. (%)	10 (12.8%)	39 (14.4%)	0.55
4.5-no. (%)	3 (3.8%)	1 (0.4%)	
5-no. (%)	59 (75.6%)	224 (82.7%)	
6-no. (%)	6 (7.8%)	7 (2.5%)	

Continuous variables are expressed as mean and standard deviation. Categorical variables are presented as numbers and percentages.

*DOAC* direct oral anticoagulant.

## Discussion

This study primarily found that the hemostasis success rate in patients who used the VasoSTAT in 4 h was not significantly different from that of those who used the TR Band in 6 h. Further, ACTs were predictive of hemostasis, and good control of ACT led to improved hemostasis success rates.

TRI has become an increasingly common PCI procedure in recent years, with endovascular therapy (EVT) also being performed by TRI. Therefore, it is widely acknowledged that the number of patients undergoing TRI continues to increase. Regardless of PCI and EVT, adequate hemostasis is achieved using the VasoSTAT.

The blood circulation in the puncture site is better with the use of VasoSTAT than TR Band; hence, primary and secondary hemostasis are readily obtained with the use of the former. Therefore, hemostasis time is shorter and the success rate is higher with the use of VasoSTAT than TR Band. A study by Safirstein et al. indicated that the VasoSTAT could significantly shorten hemostasis time by 34 min compared with the TR Band (208 min vs. 242 min), especially in PCI patients (by 43 min, 221 min vs. 264 min)^8^. We set the hemostasis time as 4 h in our study, which was appropriate. The VasoSTAT also has several other general advantages. It does not compress surrounding vessels and nerves and can, therefore, reduce pain and numbness. Furthermore, a decompression protocol is unnecessary. As a result of these effects, patients and medical staff experience low stress levels. A study by Minor et al. indicated that the VasoSTAT could significantly increase patient-reported comfort compared with the TR Band^9^. The VasoSTAT could also stop bleeding by minimal pressure and results in a low rate of RAO, as demonstrated by previous studies^8–10^. The short duration of hemostatic compression^11^ and non-occlusive compression of the puncture site^12,13^ were the causes for decreased rates of early and chronic RAO, owing to the ability of VasoSTAT to lower the rate of RAO. Pancholy et al. reported that ipsilateral ulnar artery compression during radial artery hemostasis improved blood circulation through the radial artery, which reduced the incidence of RAO, compared with only radial compression in patients undergoing diagnostic cardiac catheterization^14,15^. However, Safirstein et al. reported that ulnar artery compression with simultaneous radial artery hemostasis did not improve hand perfusion and that the rate of RAO was not significantly different between the VasoSTAT and TR Band group patients^8^. In this study, we did not compress the ulnar artery or evaluate the circulation of the hand, although the rate of RAO was significantly lower in the VasoSTAT group than in the TR Band group patients. Further prospective studies may be necessary to evaluate the relationship between blood circulation and RAO, and the hemostasis device and RAO in patients undergoing PCI.

In this study, ACT was found to be an indicator of adequate hemostasis. ACT can be easily measured in a laboratory. It is recommended that ACT be maintained between 250 and 400 s during the PCI procedure^16,17^. By measuring it from the start to the end of the PCI procedure, PCI safety and adequate hemostasis can be ensured. Patient age was not found to be significantly different between the hemostasis success and failure groups but tended to be higher in the latter. In general, due to the fragility of blood vessels and increased prevalence of hypertension, age is regarded as a risk factor for bleeding, as it is in patients undergoing PCI^18,19^. Therefore, it is necessary to be watchful of bleeding interventional procedures in elderly patients.

Distal TRI (d-TRI) is less invasive at the puncture site than TRI^20^, as a result of which, the number of d-TRI cases has increased. A previous study reported that the use of VasoSTAT in d-TRI and tibiopedal artery resulted in adequate hemostasis^10^; thus, it may be the procedure of choice in these cases. Further studies are necessary to determine whether our hemostatic protocol applies to these cases as well.

This study has several limitations. First, this was a non-randomized, retrospective study. Second, this study was performed in a single center with a small sample size; therefore, the possibility of operator bias cannot be excluded. Third, owing to the prolonged study time, the possible contributive role of the operator’s learning curve cannot be excluded. In the post-protocol group, the success rate was 89.5% from May 2017 to March 2018, 92.5% from April 2018 to March 2019, and 96.1% from April 2019 to March 2020.

In conclusion, adequate hemostasis can be achieved in lesser time (4 h vs. 6 h) using the VasoSTAT than using the TR Band. ACT can help indicate adequate hemostasis and, therefore, can be controlled to achieve an improved hemostasis success rate.

## Data Availability

The datasets generated during and/or analyzed during the current study are available from the corresponding author on reasonable request.
